# Cholesterol–PEG comodified poly (*N*-butyl) cyanoacrylate nanoparticles for brain delivery: *in vitro* and *in vivo* evaluations

**DOI:** 10.1080/10717544.2016.1233590

**Published:** 2017-02-03

**Authors:** Xiao Hu, Feifei Yang, Yonghong Liao, Lin Li, Lan Zhang

**Affiliations:** 1 Department of Pharmacology, Xuanwu Hospital of Capital Medical University, Beijing Institute for Brain Disorders, Key Laboratory for Neurodegenerative Diseases of Ministry of Education, Beijing, China and; 2 Institute of Medicinal Plant Development, Chinese Academy of Medical Sciences and Peking Union Medical College, Beijing, China

**Keywords:** Cholesterol, blood–brain barrier, brain delivery, poly (*N*-butyl) cyanoacrylate nanoparticles, PEG

## Abstract

This study investigated cholesterol–polyethylene glycol (PEG) comodified poly (ethyleneglycol)-poly (lactide) nanoparticles (CLS-PEG NPs) as a novel, biodegradable brain drug delivery system and included an evaluation of its *in vitro* and *in vivo* properties. To this end, coumarin-6 (C6), a fluorescent probe, was encapsulated into CLS-PEG NPs by an emulsion polymerization method. We reported that the use of CLS-PEG NPs led to a sustained drug release *in vitro*. Additionally, cell viability experiments confirmed their safety. The uptake and transport of CLS-PEG NPs, by bEnd.3 cells (an immortalized mouse brain endothelial cell line), was significantly higher than that of a control C6 solution. An investigation of the uptake mechanisms of different NP formulations demonstrated that cholesterol modifications may be the primary way to improve the efficiency of cellular uptake, wherein macropinocytosis may be the most important endocytic pathway in this process. An investigation of the transport mechanisms of CLS-PEG NPs also implicated macropinocytosis, energy and cholesterol in bEnd.3 cells lines. Following an intravenous (IV) administration to rats, pharmacokinetic experiments indicated that C6-loaded CLS-PEG NPs achieved sustained release for up to 12 h. In addition, IV delivery of CLS-PEG NPs appeared to significantly improve the ability of C6 to pass through the blood–brain barrier: the concentration of C6 found in the brain increased nearly 14.2-fold when C6 CLS-PEG NPs were used rather than a C6 solution. These *in vitro* and *in vivo* results strongly suggest that CLS-PEG NPs are a promising drug delivery system for targeting the brain, with low toxicity.

## Introduction

Central nervous system (CNS) disorders, including stroke, trauma, bacterial or viral infections, multiple sclerosis, Alzheimer's disease, Parkinson's disease, epilepsy and brain tumors, are a class of intractable diseases that represent a significant societal burden. However, most drugs designed to treat CNS disorders cannot cross the blood–brain barrier (BBB) to achieve a satisfactory therapeutic effect. In fact, more than 98% of small-molecule drugs, and almost 100% of large-molecule drugs, cannot cross the BBB (Pardridge, 2007; Kasinathan et al., 2015). Therefore, the most important challenge for the development of CNS drugs is achieving targeted delivery across the BBB and into the brain. Recent research has focused heavily on this issue, resulting in the development of various drugs and targeting strategies to overcome this barrier (Neuwelt et al., 2011; Fan & Yeh, 2014; Mahajan et al., 2014; Fan et al., 2015). However, there remain a number of challenges and unresolved issues that require further research.

Recently, the development of nanoparticles (NPs) has received a great deal of attention, as promising carrier systems for the delivery of CNS drugs to the brain. Specifically, NPs have a high drug loading capacity, exhibit stability *in vitro* or *in vivo*, achieve controlled release and are easily produced in an industrial setting (Bhaskar et al., 2010; Md et al., 2013; Simsek et al., 2013; Zhou et al., 2013; Jain et al., 2016). Among them, poly (*N*-butyl cyanoacrylate) (PBCA) NPs, which are coated with polysorbate 80, show tremendous potential as a brain delivery system, owing to their biodegradability and biocompatibility, as well as their bioabsorbable, bioadhesive and nonimmunogenic characteristics and have been widely investigated for controlled and targeted drug delivery (Joshi et al., 2010; Yordanova et al., 2013; Kolter et al., 2015). Multiple studies have reported that drugs (e.g. growth factors, antiepileptics and chemotherapeutic agents) that normally cannot cross the BBB can be transported across by encapsulating the drug in PBCA NPs (Kreuter et al., 1995; Alyautdin et al., 1997,1998; Friese et al., 2000; Koffie et al., 2011; Tian et al., 2011; Lin et al., 2012). However, these NPs have some limitations: (1) they may lead to an increase in brain polysorbate 80 concentrations, which may cause serious side effects, and (2) they are easily identified, and nonspecifically swallowed by macrophages in the reticuloendothelial system (including the liver and spleen) after intravenous administration *in vivo*, which shortens residence times and results in poor brain penetrance.

To penetrate the BBB and overcome macrophage clearance, a variety of modifications have been tested, resulting in the development of a dual-modified drug delivery system based on cholesterol and polyethylene glycol (PEG). This dual-modified strategy involves the modification of nanocarriers with two kinds of material: one of which (cholesterol) can cross the BBB, and the other (PEG) can avoid macrophage clearance (Li et al., 2011; Xia et al., 2012; Gutowski et al., 2015; Liu et al., 2015; Xu et al., 2015). Additionally, since PEG is approved by the FDA, and cholesterol is an endogenous material, this system is considered safe.

Therefore, the main objective of this study was to develop a novel system, using cholesterol-PEG dual modified poly (ethyleneglycol)-poly (lactide) NPs (CLS-PEG NPs) and characterize its efficacy in targeted brain delivery. To this end, coumarin-6 (C6), which is a lipophilic fluorescent probe, was utilized as a model probe. The cytotoxicity of CLS-PEG NPs was investigated in bEnd.3 cells, as well as the cellular uptake and transport mechanisms associated with them. Finally, we included an analysis of the pharmacokinetics and tissue distribution of CLS-PEG NPs.

## Materials and methods

### Materials

The C6, PEG_20000_, cholesterol (water soluble), polysorbate 80, Dextran (70 kDa Mr) and inhibitors, including chlorpromazine (CPZ), Genistein, NaN3, methyl-β-cyclodextrin (MβCD), Brefeldin A, cytochalasin D and nystatin, used in this study were all purchased from Sigma-Aldrich (St. Louis, MO). Leucine was purchased from Alfa Aesar (MA). L-aspartic acid 1-benzyl ester was purchased from Aladdin Chemistry Co., Ltd (Shanghai, China). *N*-Butyl cyanoacrylate was purchased from Shun-Kang Biotechnology Co., Ltd. (Beijing, China). Water was purified by a Milli-Q water purification system (Millipore, Bedford, MA). All other chemicals and reagents were of analytical grade.

### Preparation and characterization of PBCA NPs

#### Preparation of single-modified PBCA NPs

NPs were synthesized using an emulsion polymerization method. Briefly, dextran (70 kDa Mr) was dissolved in hydrochloric acid (pH 1) with constant stirring, using a magnetic bar. *N*-Butyl cyanoacrylate was then added in single drops to obtain a 1% vol/vol NP suspension, and stirring continued for 4 h at 500 rpm. Next, 1% (w/v) C6 was added and the stir speed was increased to 750 rpm for 2.5 h, to facilitate NP formation. Finally, the mixture was neutralized with sodium hydroxide (0.1 N) and stirred for an additional 1 h. NPs were filtered through a 0.45-μm nylon membrane, and the filtrates were dialyzed to remove unreacted materials and freeze-dried. Lyophilized NPs were resuspended in phosphate-buffered saline (PBS) with (1% w/v) cholesterol, polysorbate 80, leucine, and L-aspartic acid 1-benzyl ester or lecithin and stirred for another 30 min. The single-modified PBCA NPs were dialyzed to remove unreacted materials and freeze-dried.

#### Preparation of CLS-PEG NPs

Dextran (70 kDa Mr) and PEG_20000_ (1.5% w/v) were dissolved in hydrochloric acid (pH 1) under constant stirring using a magnetic bar. *N*-Butyl cyanoacrylate was then added in single droplets to obtain a 1% vol/vol NP suspension, and stirring continued for 4 h at 500 rpm. Next, 1% (w/v) C6 was added and the stir speed was increased to 750 rpm for 2.5 h, to facilitate NP formation. The mixture was neutralized with sodium hydroxide (0.1 N) and stirred for an additional 1 h. The NPs were filtered through a 0.45-μm nylon membrane, and the filtrates were dialyzed to remove unreacted materials and freeze-dried. Lyophilized NPs were resuspended in PBS buffer with cholesterol (1% w/v) and stirred for another 30 min. The CLS-PEG NPs were dialyzed to remove unreacted materials and freeze-dried.

#### Measurements of morphology, particle size, zeta potentials, drug loading capacity and encapsulation efficiency

The particle size (hydrodynamic diameter) and zeta-potential of the NPs were measured by dynamic light scattering using a Zetasizer Nano ZS (Malvern Instruments Ltd., UK). The experiments were performed in triplicate. The drug-loading content (DL) and encapsulation efficiency (EE) of NPs were determined by a fluorescence microplate reader (Fluoroskan Ascent FL, Thermo Fisher Scientific, Waltham, MA) at excitation and emission wavelengths of 430 nm and 538 nm, respectively. Transmission electron microscopy (TEM) was used to characterize NPs for size and particle morphology (TEM, JEM-1400, JEOL, Japan). Briefly, a drop of NP solution was placed on a copper grid and stained with 1% (w/v) of phosphotungstic acid. After the grid surface was air-dried, it was observed on the electron microscope.

#### Determination of surface concentration of cholesterol in CLS NPs and CLS-PEG NPs

The average surface concentration of cholesterol modified per NPs was calculated by dividing the weight of cholesterol absorption on the surface (using cholesterol quantitation kit (Sigma-Aldrich, St. Louis, MO)) by the calculated average number (*N*) of NPs using the following equation (Olivier et al., 2002):

$$N=\frac{6\times W\times 10^{-3}}{\pi \times {\left(D\times 10^{-7}\right)}^{3}\times \rho }$$

where *W* is the NPs weight, *D* the number based mean NPs diameter determined by QELS, ρ the NPs weight per volume unit (density), estimated to be 1.1 g/cm^3^.

#### In vitro release

One milliliter of NPs, at a C6 concentration of 50 μg/mL, was pipetted into a dialysis bag (Spectra/Por MWCO 8000–14,000 Da), which was placed in 100 mL of PBS buffer (pH 7.4), under continuous 100 rpm magnetic stirring at 37 °C. Samples (100 μL) were collected at different time points and immediately replenished with 100 μL of fresh, preheated PBS. The amount of C6 was determined by a fluorescence microplate reader as described earlier.

#### Brain distribution studies

Wistar rats (180–200 g) were obtained from the Institute of Laboratory Animal Science, Chinese Academy of Medical Sciences, Beijing, China, and all animal experiments were performed in accordance with the regulations of the Animal Care and Use Committee of the Chinese Academy of Medical Sciences. The rats were housed in a 12-h light/12-h dark cycle, at a controlled temperature of 22 °C in a specific pathogen-free (SPF) animal house. A standard diet of food and water was supplied *ad libitum* during the experiments.

The rats were divided into eight groups. Depending on their group, rats were administered either a C6 solution, PBCA NPs, single-modified PBCA NPs (modified with cholesterol, polysorbate 80, leucine, L-aspartic acid 1-benzyl ester, or lecithin) or CLS-PEG NPs via an IV administration, at a dose of 0.5 mg/kg. At 0.5 h after dosing, three rats were euthanized by cervical dislocation, and their brain were collected, washed and weighed. The samples were stored at −20 °C until further analysis.

### *In vitro* safety evaluation of C6 CLS-PEG NPs

#### Cell culture

BEnd.3 cells are an immortalized mouse brain endothelial cell line and were procured from Su-Er Biotechnology Co., Ltd. (Shanghai, China). Cells were cultured in DMEM supplemented with 10% fetal bovine serum, 100 units/mL of penicillin, and 100 μg/mL of streptomycin at 37 °C, in a humidified incubator containing 5% CO_2_.

#### In vitro cytotoxicity of C6 CLS-PEG NPs on bEnd.3 cells

To evaluate the cytotoxicity of CLS-PEG NPs *in vitro*, cell viability was evaluated by MTT assays. The concentration of samples from 5 to 200 μg/mL was incubated with the cells for 24 h, or 48 h at 37 °C. Cells without the addition of samples were used as a control group. Five replicates were included in each group.

#### In vitro hemolysis

To test hemolysis, blood was taken from the vein of rabbit ear margin. After the hemaleucin in the whole blood of rabbit was discarded, the blood was washed three times with physiological saline solution. The washed erythrocytes suspended in physiological saline solution in a final concentration of 2% (Khatik et al., 2016; Li et al., 2016). The erythrocyte suspension (0.5 mL) in physiological saline solution was mixed with 0.5 mL of CLS-PEG NPs, to obtain concentrations of 200, 100, 50, 20, 10 and 5 μg/mL and the mixture was incubated at 37 °C for 1 h in a shaker under gentle agitation. After incubation, the reaction mixture was centrifuged at 3000 r/min for 10 min and the absorbance at 570 nm of the supernatant was read in a spectrophotometer at 540 nm. As a negative hemolysis control (0% hemolysis), erythrocytes were incubated in physiological saline solution in the same condition as the samples. As a positive control to determine the value of 100% hemolysis, erythrocytes were incubated with distilled water, also in the same experimental condition. All the tests were performed in triplicate. The percentage of hemolysis was calculated from the measured absorbance values by using the relation:

$$\mathrm{Hemolysis}\;\left(\%\right)=\frac{A_{\mathrm{sample}}-A_{\mathrm{negative}\;\mathrm{control}}}{A_{\mathrm{positive}\;\mathrm{control}}-A_{\mathrm{negative}\;\mathrm{control}}}\times 100\%$$

### The uptake of C6 CLS-PEG NPs by bEnd.3 cells

#### The uptake of C6 CLS-PEG NPs by bEnd.3 cells

BEnd. 3 cells were seeded into a 6-well plate and cultured for 24 h. Then, the medium was replaced with different concentrations (20–100 μg/mL) of free C6 or CLS-PEG NPs encapsulated with C6, and incubated at 37 °C for 2 h. To study the effects of incubation time on CLS-PEG NPs uptake, the CLS-PEG NPs loaded C6 (50 μg/mL) and the free C6 solution were added into the wells, and the plate was incubated at 37 °C for 30 min, 1 h, 2 h, or 4 h. At the end of the experiment, the cells were washed with ice-cold PBS three times. Subsequently, the cells were lysed with cell lysate (Beijing Saichi Biotechnology Co., Ltd., China). The quantitative measurement of intracellular C6 in bEnd.3 cell lysates was analyzed by a fluorescence microplate reader as described earlier. Total cell protein concentrations of the bEnd.3 cells were determined using a BCA protein assay (Beijing Kangwei Century Biotechnology Co., Ltd., China). The uptake of CLS-PEG NPs by bEnd.3 cells was calculated and expressed as the amount of CLS-PEG NPs (μg) taken up per mg of cell protein.

#### Mechanistic studies on the uptake of C6 CLS-PEG NPs

A number of inhibitor treatments were used in the inhibition experiments, all of which, along with their respective functions and concentrations, are listed in Table S1. For these experiments, bEnd. 3 cells were seeded into a 6-well plate and cultured for 24 h. Then, the inhibitors were pretreated into the wells and incubated at 37 °C for 30 min, followed by incubation at 37 °C for another 2 h, after the addition of 50 μg/mL normal NPs, cholesterol single-modified PBCA NPs (CLS NPs) or CLS-PEG NPs. Blanks were prepared by adding NPs alone. At the end of the experiment, the cells were washed with ice-cold PBS three times. Subsequently, the cells were lysed with cell lysate (Beijing Saichi Biotechnology Co., Ltd., China). The quantitative measurement of intracellular C6 in bEnd.3 cell lysates was analyzed by a fluorescence microplate reader as described earlier. Total cell protein concentrations in the bEnd.3 cell were determined using a BCA protein assay (Beijing Kangwei Century Biotechnology Co., Ltd., China). The uptake of NPs by bEnd.3 cells was calculated and expressed as the amount of NPs (μg) taken up per mg of cell protein.

### Demonstration of the transport of CLS-PEG NPs across the bEnd.3 cells monolayer

#### Transport of C6 CLS-PEG NPs across the cell monolayer

First, culture media were removed from the mature bEnd.3 cell monolayers, and the monolayers were washed three times with HBSS at 37 °C. After incubation with HBSS for 30 min, CLS-PEG NPs were added to the apical chamber at a concentration of 20, 50 or 100 μg/mL. After 15, 30, 60, 90 and 120 min, 200 μL samples from the basolateral chamber were removed, and the same volume of fresh and preheated HBSS was added. The samples were centrifuged at 12 000 rpm for 5 min. Finally, the amount of C6 in the supernatant was analyzed by a fluorescence microplate reader as described earlier.

The *P*_app_ value reflected the ability, absorption rate, and extent of drug transport and is expressed as cm/s. The *P*_app_ value was obtained by the following formula:

$$P_{\mathrm{app}}=\frac{\mathrm{dC}}{\mathrm{dt}}\times \frac{V}{A\times C_{0}}$$

where *A* is the surface area of the cell monolayers (1.12 cm^2^ in this study), *C*_0_ is the initial concentration of C6 (μg/mL) in the apical chamber, and dQ/dt is the linear appearance rate in the basolateral chamber.

#### Mechanistic studies on the transport of C6 CLS-PEG NPs

The inhibitors used for the transport test are also listed in Table S1. The cell monolayers were first incubated with different inhibitors for 30 min at 37 °C, then CLS-PEG NPs (50 μg/mL) were added to the apical chamber. A control was included without inhibitors. After 15, 30, 60, 90 and 120 min, 200 μL samples from the basolateral chamber was removed, and the same volume of fresh and preheated HBSS was added. The samples were centrifuged at 12 000 rpm for 5 min. The amount of C6 in the supernatant was analyzed by a fluorescence microplate reader as described earlier. Finally, the *P*_app_ values of the CLS-PEG NPs were determined.

### Pharmacokinetic study

A polyethylene catheter (Portex, Hythe, UK) was inserted through the right jugular veins of all rats one day prior to the pharmacokinetic experiment, and flushed with 100 U/mL heparin in 0.9% saline. After at least 12 h of recovery postsurgery, the intubated rats were divided into three groups and received IV administration of free C6 solution, CLS NPs, or CLS-PEG NPs at a dose of 0.5 mg/kg.

After dosing, 200 μL of blood was collected at predetermined time points (0.083, 0.25, 0.5, 0.75, 1, 2, 4, 6, 8 and 12 h). Subsequently, 100 μL of plasma was obtained by centrifugation at 5000 rpm for 7 min at 0 °C, and the serum was collected. The measurement of C6 in the samples was analyzed by a fluorescence microplate reader as described earlier.

### Tissue distribution studies

The rats were divided into three groups and free C6 solution, CLS NPs, or CLS-PEG NPs were administered, via IV, at a dose of 0.5 mg/kg. At predetermined time points (0.25, 0.5, 1, 2, 4, 8 and 12 h) after dosing, three rats at each time point were euthanized by cervical dislocation, and their heart, liver, spleen, lung, kidney and brain were collected, washed and weighed. The samples were stored at −20 °C until further analysis.

The drug-targeting index (DTI) is considered an important parameter for tissue targeting efficiency, and was calculated using the following formula:

$$\mathrm{DTI}=\frac{\frac{{\mathrm{AUC}}_{x}}{{\mathrm{AUC}}_{p}}(\mathrm{NPs})}{\frac{{\mathrm{AUC}}_{x}}{{\mathrm{AUC}}_{P}}(\mathrm{Free}\;\mathrm{drug})}$$

Where “AUC_x_” is the AUC of tissue x, and “AUC_p_” is the AUC of the plasma.

### Determination of specificity to the brain

Before beginning the microscopic experiments, free C6 solution, NPs, CLS NPs, or CLS-PEG NPs were administered to the rats by IV, and three rats were sacrificed and cardiac perfused 0.5 h after dosing. Subsequently, the brain tissues were removed, washed with PBS, and fixed in 4% formaldehyde solution. After snap freezing in liquid nitrogen, the samples were embedded in tissuetek O.C.T. compound (Sakura Finetechnical, Tokyo, Japan) and cut into thin sections (10 μm). The slices were stained for cell nuclei with DAPI for 15 min in the dark and then washed three times with cold PBS. The sample was monitored by confocal laser scanning microscopy (Zeiss LSM710, Göttingen, Germany). ZEN Image Software 2012 was used to perform the image recording and image analysis. The image included 512 × 512 pixels measuring 2.77 × 2.77 μm^2^.

### Data analysis

The data are expressed as the mean values ± standard deviations (SD). Statistical analysis was performed using SPSS software, version 17.0 (SPSS Inc., Chicago, IL). The data were analyzed by a Student’s two-tailed *t*-test or a factorial analysis of variance (ANOVA), followed by *post hoc* comparisons using Dunnett’s test, or the Student–Newman–Keuls (SNK) test if the ANOVA manifested a significant difference. *p < 0.05 was considered statistically significant.

## Results and discussion

### Characterization of PBCA NPs

#### Measurements of morphology, particle size, zeta potentials, drug loading capacity, and encapsulation efficiency

The characterizations of the NP systems are shown in Table S2. As shown in Table S2, the modifications to the surface of the PBCA NPs may have influenced particle size, especially in the case of lecithin. In addition, the NPs exhibited excellent DL and EE; the DL of C6 for the NPs was about 2%, and the EE of C6 for the NPs was all higher than 96%.

The particle size of CLS NPs was determined to be 175.6 ± 4.1 nm (PDI 0.264 ± 0.017), whereas the size was increased to 185.4 ± 4.1 nm (PDI 0.133 ± 0.009) in the CLS-PEG NPs (Figure S1a). Additionally, the zeta-potential of CLS NPs and CLS-PEG NPs were −28.5 ± 1.43 mV and −0.66 ± 0.10 mV (Figure S1b), respectively. These results show that the CLS-PEG modification did not significantly influence particle size but did significantly increase the zeta potential. Visualization with TEM showed that there was no change in morphology between the CLS NPs and CLS-PEG NPs: they were both generally spherical and of regular size (Figure S1).

#### Determination of surface concentration of cholesterol in CLS NPs and CLS-PEG NPs

The average surface concentration of cholesterol modified per CLS NPs or CLS-PEG NPs was calculated respectively, which gave a mean of (6.6 ± 0.2) × 10^−^^7 ^ng cholesterol per CLS NPs or (8.5 ± 0.4) × 10^−^ ^7 ^ng cholesterol per CLS-PEG NPs. Compared with the CLS NPs, higher surface concentration of cholesterol in CLS-PEG NPs indicated that CLS-PEG NPs might be potentially useful to brain targeting *in vivo*.

#### In vitro release

**Figure 1.**
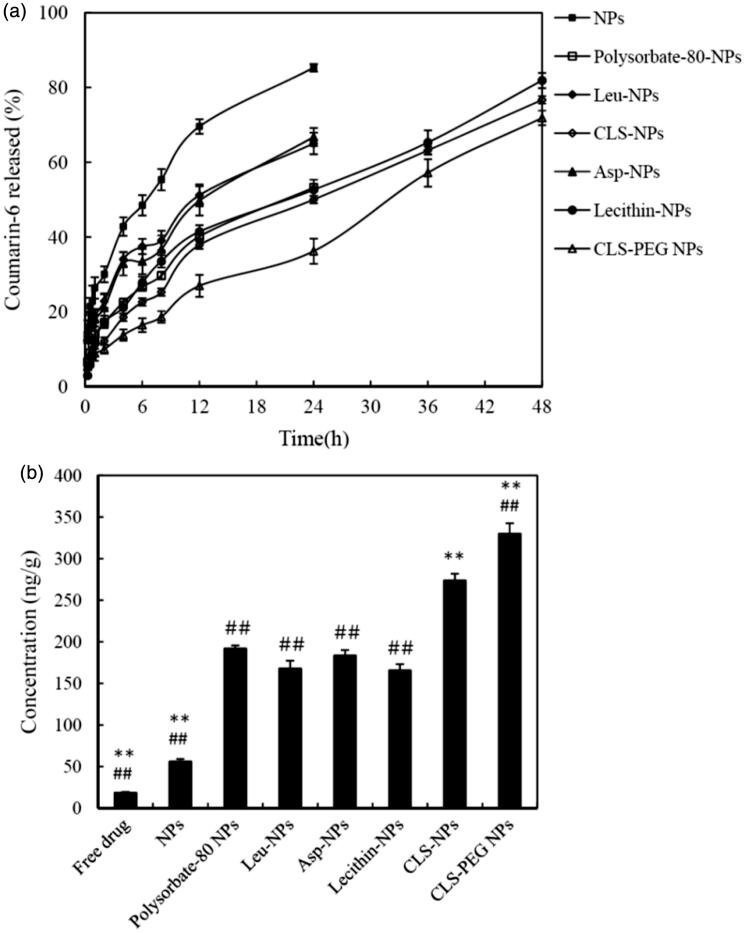
(a) The *in vitro* release profiles of NPs. (b) Mean concentration of C6 in brains after IV administration of C6 formulations to rats at a dose of 0.5 mg/kg (mean ± SD, *n* = 3). **p < 0.01 versus the polysorbate-80 NPs group. ##*p* < 0.01 versus the CLS NPs group.

The *in vitro* release profiles of the NP systems, which were evaluated by dialysis, are shown in Figure 1(a). The C6 content from CLS-PEG NPs was significantly higher than that from the single-modified PBCA NPs (ANOVA, *p *<* *0.05), which indicates that CLS-PEG NPs were potentially useful to control the release of coumarin-6 and that the significantly sustained release behavior is likely attributed to the slower degradation of the CLS-PEG NPs rather than other NPs systems. Polyethylene glycol (PEG), a polymer of ethylene oxide monomers, being most often used for drug delivery systems as the matrices for controlled release and has been approved by Food and Drug Administration for therapeutic injections (Harris et al., 2001; Kolate et al., 2014). The drug-release rate in the medium depends on the degree of degradation of the NPs, which can be changed effectively by the use of some the polymer carriers. The CLS-PEG NPs may possess more reactive coupling dots and restrain chain movements. The interaction force among the polymer chains is strengthened, which does not facilitate water attack, and thus impairs the rate of hydrolysis (Stefani et al., 2006; Luo et al., 2011; Fasehee et al., 2016). Due to degradation of the CLS-PEG NPs, the coumarin-6 released from the CLS-PEG NPs slowly, and then, the free coumarin-6 can be introduced into the medium via dissolution or diffusion. So the least coumarin-6 accumulative release in CLS-PEG NPs among all NP systems might be due to the affect of PEG on the degradation of CLS-PEG NPs.

#### Brain distribution studies

The brain distribution data for the C6 formulations after IV administration are shown in Figure 1(b). The results show that the concentration of C6 in the brain was significantly higher in rats treated with modified NPs, than in those treated with free drug or normal NPs (*p < *0.01). Moreover, brain targeting of the NPs coated with CLS was significantly increased as compared to other modifications. Furthermore, the brain distribution studies found that the concentration of C6 was significantly higher in the brains of rats treated with CLS-PEG NPs, than of those treated with CLS NPs (*p < *0.01). These results demonstrate that modification of PBCA NPs with cholesterol and PEG might increase the efficiency of brain delivery.

### *In vitro* safety evaluation of C6 CLS-PEG NPs

#### In vitro cytotoxicity of C6 CLS-PEG NPs on bEnd.3 cells

**Figure 2.**
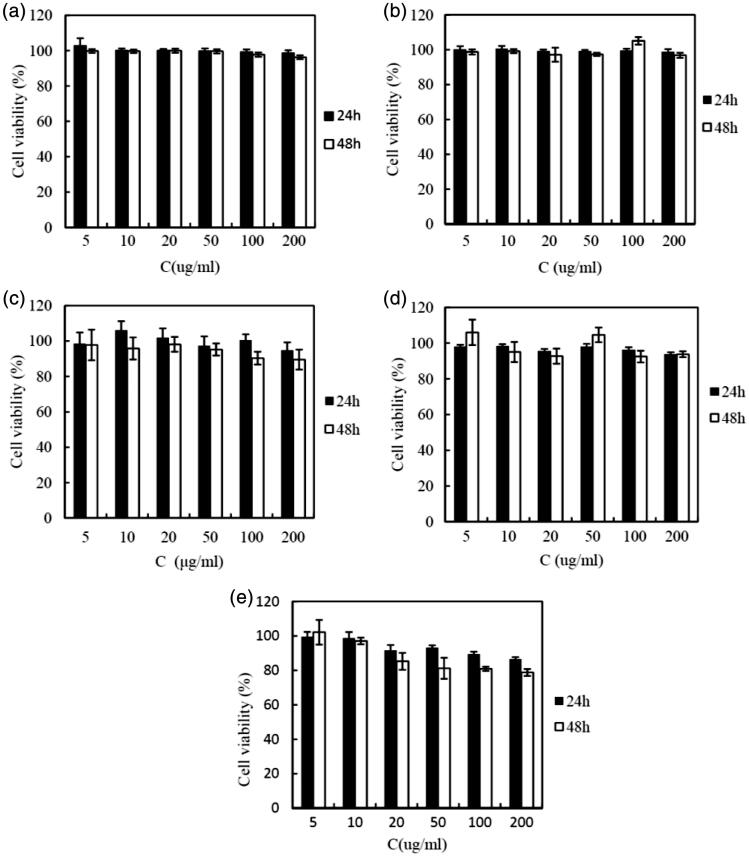
Cytotoxicity of (a) cholesterol, (b) PEG_20000_, (c) PBCA, (d) blank CLS-PEG NPs, and (e) C6 loaded CLS-PEG NPs in bEnd.3 cell line at different concentrations as assessed by MTT levels (mean ± SD, *n *= 5).

Safety is one of the most important factors that should be considered when developing new polymeric brain delivery methods. Cholesterol is generally accepted as an important, endogenous molecule, while it has been demonstrated that PEG is biodegradable and “safe” *in vivo* (Zambaux et al., 2001). The PBCA used in this study has also been proved to be a biosafety carrier (Couvreur et al., 1982). Thus, the CLS-PEG NPs can be speculated as safe carriers. To confirm this, MTT assays were performed to evaluate their cytotoxicity in bEnd.3 cell lines. Cells without treatment with a carrier were used as a control and had a cell viability of 100%. As shown in Figure 2, the polymer material and CLS-PEG NPs without drug did not cause cytotoxicity (cell viabilities all exceeded 95%). After treatment with C6-loaded CLS-PEG NPs, cell viability was still over 80%. Based on this data, we conclude that CLS-PEG NPs are a nontoxic and biocompatible vector.

#### In vitro hemolysis

Assessment of the hemolytic potential against a suspension of erythrocytes illustrated the security of CLS-PEG NPs *in vivo*. The hemolysis of erythrocytes of CLS-PEG NPs was < 5% after incubated at 37 °C for 1 h. The small hemolytic effect of CLS-PEG NPs suggested that the formulation could be safety for intravenous administration.

### The uptake of C6 CLS-PEG NPs by bEnd.3 cells

#### The uptake of C6 CLS-PEG NPs by bEnd.3 cells

BEnd.3 cells are an immortalized mouse brain endothelial cell line exhibiting endothelial properties. These cells are a suitable model of the BBB due to their rapid growth, maintenance of BBB characteristics over repeated passages, formation of functional barriers and amenability to numerous molecular interventions (Brown et al., 2007). Based on this, bEnd.3 cells were chosen as the BBB model used in this study to examine the uptake and transport mechanisms of CLS-PEG NPs for brain delivery *in vitro*.

**Figure 3.**
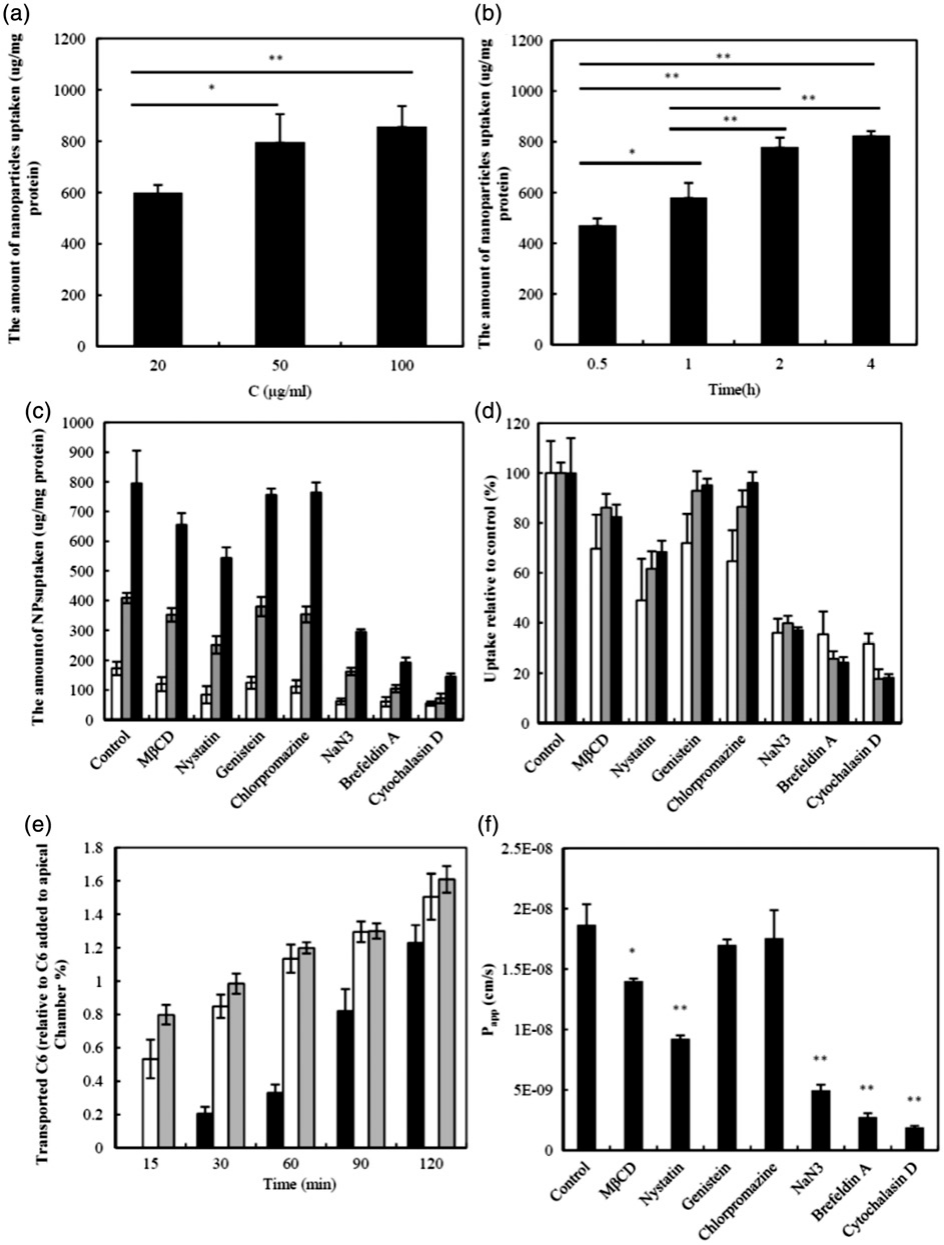
Effect of concentration (a) or the incubation time (b) on the cellular uptake of CLS-PEG NPs; Uptake (c) or uptake relative to control (d) of normal NPs (□), CLS NPs 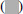 or CLS-PEG NPs (▪) by bEnd.3 cells in the presence of different inhibitors at 37 °C for 2 h; (e) Transport of C6 CLS-PEG NPs with the concentration of 20 μg/mL (▪), 50 μg/mL (□) or 100 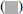 μg/mL across bEnd.3 cell monolayer at 37 °C by monitoring quantitatively the C6 in the basolateral chamber as the function of time and concentration; (f) *P*_app_ of C6 CLS-PEG NPs transport across bEnd.3 cell monolayer in the presence of different inhibitors (mean ± SD, *n *= 3). *p < 0.05, **p < 0.01 versus the control group.

The results of the cellular uptake experiments are shown in Figure 3. Uptake of CLS-PEG NPs was significantly dependent on both concentration and time; however, the uptake of CLS-PEG NPs barely increased at the highest concentration (Figure 3(a)). The same pattern was observed with respect to incubation time. Specifically, the uptake of CLS-PEG NPs by bEnd.3 cells was dependent on the incubation time, within 2 h (Figure 3(b)), but the uptake of CLS-PEG NPs rarely increased after the 2-h incubation. Taken together, these results suggest that this process involves receptor-mediated endocytosis. Additionally, we found that the free C6 solution only minimally entered the cells, similar to previous reports (Dong & Feng, 2005). These findings visually demonstrate that CLS-PEG NPs may be able to significantly increase the cellular uptake of drugs, and may be an efficient delivery method for drug treatments of CNS disorders.

#### Mechanistic studies on the uptake of C6 CLS-PEG NPs

The uptake of normal NPs, CLS NPs or CLS-PEG NPs with inhibitors is shown in Figure 3(c) and (d). The data indicate that the cellular uptake of CLS-PEG NPs was significantly higher than that of the NPs or CLS NPs, which demonstrates that the dual-modified drug delivery systems were conducive to uptake. Furthermore, the uptake of CLS-PEG NPs was significantly inhibited by cytochalasin D (macropinocytosis inhibitor), brefeldin A (disrupts the Golgi apparatus and intracellular trafficking), NaN3 (energy depletion agent), and nystatin (cholesterol inhibitor). With respect to cytochalasin D and brefeldin A, these results suggest an involvement of macropinocytosis and the Golgi apparatus in the cellular uptake of CLS-PEG NPs. Conversely, nystatin can bind to cholesterol and disrupt lipid rafts by directly inserting into membranes and sequestering cholesterol into complexes (Ushio-Fukai et al., 2013). Therefore, these results also demonstrate that cellular uptake of CLS-PEG NPs was highly cholesterol dependent. In addition, the inhibitory effect of NaN3 on the internalization of CLS-PEG NPs suggests that in addition to endocytosis, an energy-independent internalization mechanism might also be involved.

We found that the CLS NPs and CLS-PEG NPs had similar cellular uptake mechanisms (macropinocytosis may be the most important endocytic pathway in the process of cellular uptake), which was different from that of the normal NPs. These results demonstrate that cholesterol may be the primary factor that improves the efficiency of the cellular uptake of NPs and that macropinocytosis, the Golgi apparatus, energy and cholesterol might play an important role in the cellular uptake mechanisms of CLS-PEG NPs by the bEnd.3 cell lines. However, not caveolae- and clathrin-mediated endocytosis pathway were involved, wherein macropinocytosis may be the most important endocytic pathway in the process of cellular uptake of CLS-PEG NPs.

### Demonstration of the transport of NPs across the bEnd.3 cell monolayers

#### Transport of C6 CLS-PEG NPs across the cell monolayer

The transport of CLS-PEG NPs across the bEnd.3 cell monolayer, at 37 °C, is shown in Figure 3(e). We report a positive correlation of transport with time and concentration; specifically, the higher the concentration, the more C6 transportation was observed as time went on. However, at the high concentration, transport was barely enhanced by the concentration. The *P*_app_ values for the CLS-PEG NPs, with concentrations of 20, 50 or 100 μg/mL, were (1.52 ± 0.13)× 10^−^ ^6^, (1.87 ± 0.17) × 10^−6^ and (2.00 ± 0.10) × 10^−^ ^6 ^cm/s, respectively. Additionally, there was no statistically significant difference in the *P*_app_ values at the high concentration. These data demonstrate that the transport mechanisms of CLS-PEG NPs may be mediated by endocytosis.

#### Mechanistic studies on the transport of C6 CLS-PEG NPs

The results of the transport studies, of CLS-PEG NPs with inhibitors, are shown in Figure 3(f). The *P*_app_ values with MβCD and nystatin were (1.40 ± 0.02) × 10^−^ ^6^ and (0.92 ± 0.03) × 10^−^ ^6 ^cm/s, respectively. Moreover, MβCD depressed transport significantly, while the intracellular retention level of C6 was not significantly different from that of the control. This finding is similar to previous reports (Zhao et al., 2011), which suggest that different mechanisms are involved in endocytosis and exocytosis. Importantly, the significant inhibition observed following treatment with MβCD and nystatin demonstrates that the endocytosis of CLS-PEG NPs was highly cholesterol-dependent.

The *P*_app_ values following treatment with NaN_3_ and brefeldin A were (0.50 ± 0.04) × 10^−^ ^6^ and (0.28 ± 0.03)× 10^−^ ^6^ cm/s, respectively. The significant inhibition observed with NaN3 and brefeldin A indicates that the endocytosis of CLS-PEG NPs was also energy and Golgi apparatus dependent. Additionally, significant inhibition resulted from treatment with cytochalasin D, with a *P*_app_ value of (0.19 ± 0.01) × 10^−^ ^6^. It has been suggested that micropinocytosis-mediated pathways are also involved and are perhaps even the most important, in the transport of CLS-PEG NPs. Taken together, these results show that macropinocytosis, the Golgi apparatus, energy, and cholesterol might play an important role in the transport mechanisms of CLS-PEG NPs in bEnd.3 cell lines.

### Pharmacokinetic study

**Figure 4.**
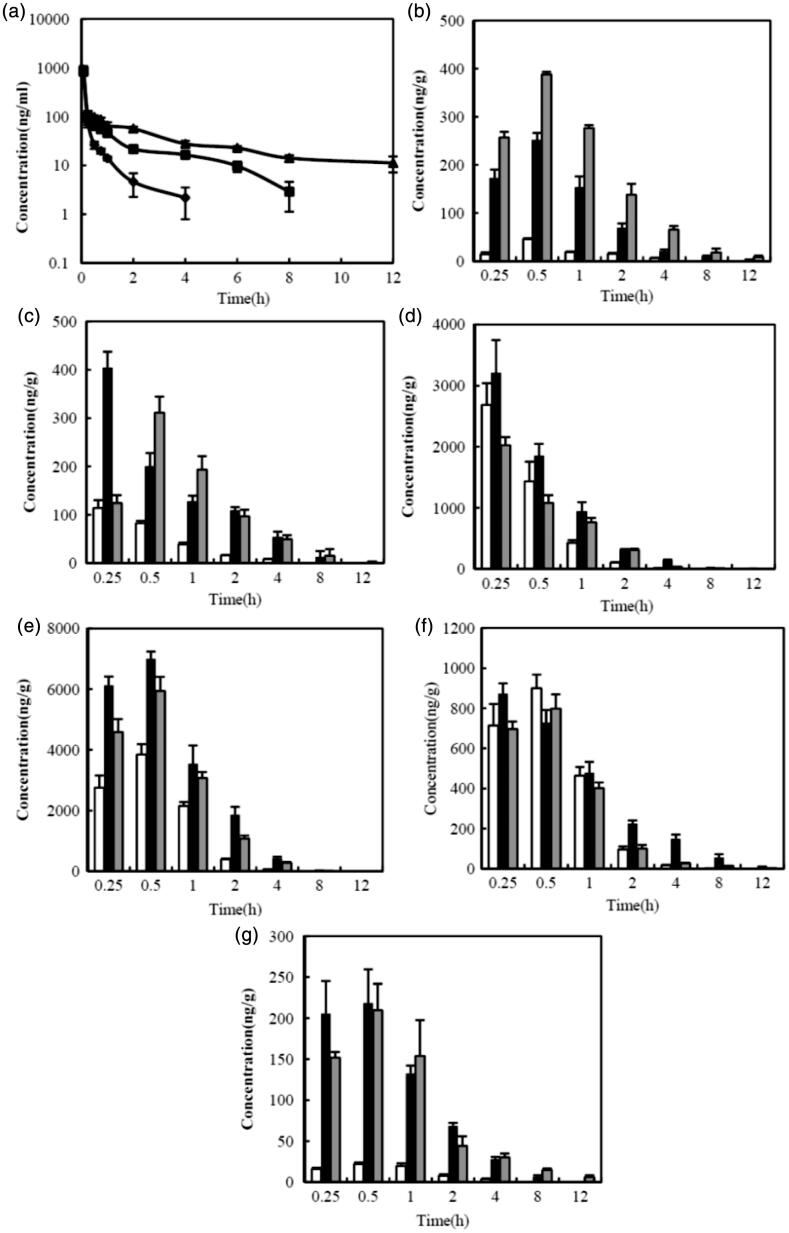
Mean concentration-time profiles of C6 in organs after IV administration of free C6 solution (♦) (□), CLS NPs (▪) (▪) or CLS-PEG NPs (▴) (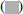) to rats at a dose of 0.5 mg/kg (means ± SD, *n* = 3). (a) plasma; (b) brain; (c) heart; (d) liver; (e) spleen; (f) lung; (g) kidney.

The C6 plasma concentration curves, with respect to time, after IV administration of NPs are shown in Figure 4(a), and the pharmacokinetic parameters are summarized in Table 1. As shown in Figure 4(a), the *C*_0_ value was not found to be significantly different between formulations in the systemic circulation. However, C6 was still present at a relatively high concentration 12 h after injection of CLS-PEG NPs, but was undetectable 8 h or 4 h after injection of CLS NPs or free drug, respectively. In addition, both NPs had higher AUC values than that of the free drug, and this was also true for the *t*_1/2_. Specifically, the *t*_1/2_ and AUC of CLS-PEG NPs were approximately 4.58- and 2.12-fold increased, compared with those of the free drug, respectively (Table 1). The present results suggest that CLS-PEG NPs can sustain C6 delivery and improve bioavailability. The prolonged presence of C6 in the CLS-PEG NPs in the plasma may be due to the PEG modification on the surface of the PBCA NPs.

**Table 1.** Pharmacokinetic parameters of C6 after the IV administration of free C6 solution, CLS NPs and CLS-PEG NPs to rats at a dose of 0.5 mg/kg (means ± SD, *n* = 6).

	Coumarin-6		
Parameters	Free drug (IV)	CLS NPs (IV)	CLS-PEG NPs (IV)
*C*_0_ (ng/ml)	828.4 ± 139.0	853.5 ± 143.8	906.6 ± 175.5
*t*_1/2_, λ_z_ (min)	60.1 ± 24.0	106.8 ± 21.7^b^	275.6 ± 117.4^b^
AUC_0-t_ (μg·min/ml)	15.8 ± 2.4	22.9 ± 3.0^b^	35.2 ± 4.4^b^
AUC_0-∞_(μg min/ml)	16.0 ± 2.3	23.3 ± 3.2^b^	39.9 ± 5.3^b^
*V*_d_, λ_z_ (l)	0.6 ± 0.3	0.6 ± 0.1	1.0 ± 0.4^a^
CL (ml/min)	6.4 ± 0.9	4.4 ± 0.6^b^	2.5 ± 0.4^b^
*F* (%)	–	144.9	212.5

^a^*p* < 0.05, ^b^*p* < 0.01 versus the free drug group.

### Tissue distribution studies

The tissue distribution data for the free drug and NP formulations, after IV administration, are shown in Figure 4, and the pharmacokinetic parameters in the tissue are summarized in Table 2. The results showed that the free solution of C6 resulted in very little distribution in the brain (Figure 4(b)), similar to previous reports showing that the BBB is considered practically impermeable to free C6 (Bhattacharyyaa et al., 2011). At all included time points, the C6 concentration in the brain was significantly higher in rats treated with CLS-PEG NPs than in those treated with the free solution or CLS NPs (*p < *0.05). Additionally, the AUC_0-t_ values of C6 in CLS-PEG NPs were 14.2- and 2.0-fold higher than those in the free drug and CLS NPs, respectively, while the *C*_max_ values of CLS-PEG NPs were 8.5- and 1.6-fold higher than those in the free drug and CLS NPs, respectively (Table 2). The brain DTI values for CLS-PEG NPs and CLS NPs were 6.46 and 5.04, respectively. These results demonstrate that modification of the PBCA NPs with cholesterol and PEG might increase the efficiency of delivery to the brain.

**Table 2.** Pharmacokinetic parameters of C6 in tissues and drug targeting index (DTI) to brain in three groups of rats after IV administration of free C6 solution, CLS NPs and CLS-PEG NPs at a dose of 0.5 mg/kg (means ± SD, *n* = 3).

Tissues	AUC_0-t_ (μg·min/ml)	*C*_max_ (ng/g)	*T*_max_ (h)	DTI
*Brain*				
Free drug	3.84 ± 0.21	45.64 ± 2.25	0.5	–
CLS NPs	27.69 ± 1.38^b^	250.70 ± 15.79^b^	0.5	5.04
CLS-PEG NPs	54.60 ± 4.21^b^	388.01 ± 5.41^b^	0.5	6.46
*Heart*				
Free drug	8.45 ± 0.93	113.89 ± 16.48	0.25	–
CLS NPs	43.11 ± 3.58^b^	403.73 ± 33.75^b^	0.25	3.52
CLS-PEG NPs	39.13 ± 1.12^b^	311.19 ± 33.38^b^	0.5	2.08
*Liver*				
Free drug	141.71 ± 13.25	2685.53 ± 356.67	0.25	–
CLS NPs	232.29 ± 16.69^b^	3209.88 ± 535.98	0.25	1.13
CLS-PEG NPs	152.28 ± 6.76	2024.32 ± 134.43^a^	0.25	0.48
*Spleen*				
Free drug	271.02 ± 16.03	3848.83 ± 339.27	0.25	–
CLS NPs	659.91 ± 33.44^b^	7003.94 ± 236.76^b^	0.50	1.68
CLS-PEG NPs	491.57 ± 4.49^b^	5940.80 ± 464.70^b^	0.5	0.81
*Lung*				
Free drug	63.88 ± 5.62	900.02 ± 67.86	0.5	–
CLS NPs	120.67 ± 4.82^b^	872.21 ± 52.16	0.25	1.30
CLS-PEG NPs	63.93 ± 1.50	797.77 ± 71.77	0.5	0.45
*Kidney*				
Free drug	2.56 ± 0.44	22.20 ± 1.91	0.5	–
CLS NPs	25.96 ± 1.54^b^	218.55 ± 40.97^b^	0.5	7.08
CLS-PEG NPs	27.75 ± 4.00^b^	209.55 ± 32.30^b^	0.5	4.93

^a^*p* < 0.05, ^b^*p* < 0.01 vs. the free drug group.

Additionally, we found that the DTI for the CLS-PEG NPs was significantly lower than for the CLS NPs in other tissues, and even significantly lower than that of the free drug in the liver, spleen, and lung (Table 2). These results indicate that the CLS-PEG NPs avoid phagocytosis by macrophage and that the CLS-PEG NPs can achieve a high total concentration in the brain, without markedly increasing accumulation in non-targeted tissues. As indicated in the study, the dual modified PBCA NPs could increase the accumulation of C6 in the brain.

To further confirm the translocation of CLS-PEG NPs across the BBB, confocal microscopy was utilized to evaluate the distribution of the varying formulations in brain tissue (Figure S2). A strong green fluorescent signal was observed in the brain after treatment with CLS-PEG NPs, whereas the fluorescent signal was too weak to be distinguished in the brain after administration of free C6. The C6 fluorescent signal at the designated time point, for each of the formulations tested, was ranked as follows: C6 plus CLS-PEG NPs > C6 plus CLS NPs > C6 plus NPs > free C6.

The delivery of therapeutic levels of drugs to the brain is a major challenge, due to the BBB. Previous studies have investigated a number of possible mechanisms by which NPs could deliver drugs across the BBB (Kreuter, 2001). The researchers found that PBCA NPs do not induce nonspecific BBB disruption, but rather work in conjunction with plasma apolipoprotein E (apoE) to facilitate BBB crossing (Koffie et al., 2011). Specifically, the mechanism involves the adsorption of apoE from the plasma, making receptor-mediated transcytosis through vascular endothelial cells the likely mechanism by which PBCA NPs cross the BBB. However, the mechanism by which CLS-PEG NPs cross the BBB is not yet clear.

Research has shown that modifying PBCA NPs with PEG can prevent phagocytosis by macrophages, thereby lengthening the time in circulation. Although studies have confirmed that nearly all cholesterol in the central nervous system is locally synthesized, and that there is minimal exchange of cholesterol across the intact BBB (Dietschy & Turley, 2001), recent work has also implicated peripheral cholesterol in CNS disorders (Reed et al., 2014). The researchers speculated that cholesterol in the blood may be related to, or interact with, chemical species that can efficiently traverse the BBB. Additionally, in this study, we found that conjugating cholesterol to the surface of NPs increased the transport of drugs across the BBB, via macropinocytosis.

Therefore, modifying PBCA NPs with cholesterol might induce active transport of NPs through the BBB. The enhanced penetration of the CLS-PEG NPs into the brain observed in this study was likely due to a combined function of cholesterol and PEG.

## Conclusions

A novel brain drug delivery system was prepared and modified with cholesterol and PEG. The CLS-PEG NPs were prepared using an emulsion polymerization method and yielded a sustained drug release *in vitro*. Results of cell viability experiments confirmed the safety of utilizing CLS-PEG NPs. The cellular uptake of CLS-PEG NPs by bEnd.3 cells was shown to be significantly higher than that of the C6 solution, NPs, and CLS NPs. Additionally, our investigation of the mechanisms underlying the uptake of NPs demonstrated that cholesterol may be the primary factor by which the efficiency of cellular uptake can be improved, wherein macropinocytosis may be the most important endocytic pathway in this process. Our results also demonstrated that macropinocytosis, energy, and cholesterol may play an important role in the transport of CLS-PEG NPs by bEnd.3 cells lines. After IV administration of NPs in rats, pharmacokinetic experiments indicated that C6 loaded CLS-PEG NPs achieved sustained release for up to 12 h; the *t*_1/2_ and AUC values of the CLS-PEG NPs were approximately 4.58- and 2.12-fold those of the C6 that was associated to free drug, respectively. Further analysis of C6 concentrations in the brain revealed that CLS-PEG NPs led to significantly higher concentrations than the free solution or CLS NPs, and that the AUC_0-t_ values of C6 in CLS-PEG NPs were 14.2- and 2.0-fold higher than those in the free drug and CLS NPs, respectively. The brain DTI values for CLS-PEG NPs and CLS NPs were 6.46 and 5.04, respectively. In addition, the DTI of the CLS-PEG NPs was significantly lower than that of the CLS NPs, as well as the free drug levels in other tissues. Thus, the present data *in vitro* and *in vivo* suggests that CLS-PEG NPs could be used as a novel means for the delivery of drugs to the brain.

## Supplementary Material

Supplemental_Figures.docx

## Notes

**Supplementary material available online**
