# Supplementary material for: Cholesterol–PEG comodified poly (N-butyl) cyanoacrylate nanoparticles for brain delivery: in vitro and in vivo evaluations
Source: Drug Deliv. 2017 Feb 3;24(1):121–32. doi: 10.1080/10717544.2016.1233590 (PMC8241168; doi:10.1080/10717544.2016.1233590)
Supplement: Supplemental_Figures.docx [file IDRD_A_1233590_SM7896.docx]

**Legends to Supplemental Figures and Tables**

**Fig. S1.** Particle size (a) and zeta potential (b) distribution of CLS-PEG NPs. TEM images of the CLS NPs (c) and CLS-PEG NPs (d).

**Fig. S2.** The specificity to the brain sites in the rats after intravenous administration. a. free C6; b. NPs; c. CLS NPs; d. CLS-PEG NPs.

**Table.S1.** Concentrations and functions for inhibitors used in the uptake inhibition experiment.

**Table.S2**. Encapsulation parameters, dynamic particle size and zeta-potentials of PBCA nanoparticles (means ± SD, *n* = 3).

**Fig. S1.**

**Fig. S2.**

**Table S1.**

| Inhibitors | Concentration | Function |
| --- | --- | --- |
| Methyl-β-cyclodextrin | 10 mM | Depletion of cholesterol |
| Nystatin | 10 μM |  |
| Genistein | 200 μM | Interact with caveolin |
| NaN3 | 10 mM | Depletes cellular ATP |
| Chlorpromazine | 30 μM | Inhibitor of clathrin mediated |
| Brefeldin A | 20 μg/ml | Golgiapparatus breake |
| Cytochalasin D | 100 mmol/L | Actin inhibitor |

**Table S2.**

|  | NPs | Polysorbate 80-NPs | Leu-NPs | Aps-NPs | Lecithin-NPs | CLS-NPs | CLS-PEG-NPs |
| --- | --- | --- | --- | --- | --- | --- | --- |
| Drug loading (%) | 1.87 | 2.31 | 2.04 | 1.98 | 1.95 | 1.96 | 1.98 |
| Encapsulation efficiency (%) | 96.3 | 97.3 | 98.3 | 96.9 | 97.4 | 97.4 | 97.8 |
| Size (nm) | 145.2 ± 3.6 | 171.8 ± 2.5 | 167.3 ± 3.2 | 146.2 ± 2.7 | 344.2 ± 4.7 | 175.6 ± 3.6 | 185.4 ± 4.1 |
| PDI | 0.276 ± 0.031 | 0.235 ± 0.023 | 0.138 ±0.011 | 0.119 ± 0.007 | 0.252 ±0.016 | 0.264 ± 0.017 | 0.133 ± 0.009 |
| Zeta-potential (mV) | -30.9 ± 2.93 | -37.5 ± 2.19 | -9.63 ±0.72 | -6.78 ± 0.27 | -37.3 ± 1.58 | -28.5 ± 1.43 | -0.66 ± 0.10 |
